# GCN2 is activated by methyl jasmonate through GCN1 and reactive oxygen species in *Arabidopsis thaliana*

**DOI:** 10.1038/s41598-025-32799-w

**Published:** 2026-01-13

**Authors:** Daniel Rincon Diaz, Morgan E. Wynn, Emmanuel Asiedu, Teressa K. Akuoko, Ansul Lokdarshi

**Affiliations:** Department of Biology, Valdosta State University, 1500 N Patterson St, Valdosta, GA 31698 USA

**Keywords:** GCN2, GCN1, eIF2α, Methyl jasmonate, Translation, Abiotic stress, Biochemistry, Molecular biology, Plant sciences

## Abstract

Plant growth and productivity rely on rapid energy management strategies to adapt dynamic environments. Previous work in *Arabidopsis thaliana* identified a fast-regulatory switch linking cytosolic translation and reactive oxygen species (ROS) signaling, where the protein kinase general control of nonderepressible (GCN)2 is rapidly activated in response to ROS under numerous stresses and phosphorylates the eukaryotic translation initiation factor (eIF)2α as a potential stress mitigation mechanism. Here, we test the hypothesis that the Arabidopsis GCN2-eIF2α’s responses towards the plant defense hormone methyl jasmonate (MeJA) are regulated by light, ROS, and the conserved GCN2 activator protein, general control of non-derepressible 1 (GCN1). We show that eIF2α phosphorylation (P-eIF2α) under MeJA stress requires light and is suppressed by antioxidants and photosynthetic inhibitors. GCN1 is essential for this activation, as *gcn1* mutant seedlings show reduced P-eIF2α in response to MeJA. Physiologically, both *gcn1* and *gcn2* mutants exhibit enhanced sensitivity to MeJA in a primary root growth assay. Surprisingly, despite impaired signaling, *gcn2* mutants maintain wild-type-like protein synthesis rates under MeJA stress, as shown by polysome profiling and puromycin labeling. Combined, we provide fresh insights into the activation of the Arabidopsis GCN2-eIF2α module in response to MeJA stress by ROS and the GCN1 protein.

**Supplementary Information:**

The online version contains supplementary material available at 10.1038/s41598-025-32799-w.

## Introduction

Plants frequently encounter a wide range of environmental conditions that adversely affect their growth, development, and overall health. To adapt and/or survive, plants have evolved complex signaling mechanisms that operate at the molecular, biochemical, and physiological levels^1,2^. One of the key metabolic signals that lies at the center of these adaptive responses is the synthesis of phytohormones such as auxin, ethylene, methyl jasmonate, salicylic acid, and abscisic acid^3,4^. These naturally occurring compounds are critical for managing plant health under different types of environmental conditions^4^. Additionally, they regulate a wide variety of growth and developmental processes, such as seed germination, root growth, fruit ripening, and leaf senescence^5–7^.

Methyl jasmonate (MeJA), a volatile derivative of jasmonic acid (JA), plays a central role in coordinating plant responses to abiotic stresses such as drought, salinity, and high light, as well as biotic challenges including herbivory and pathogen attack^5,6,8^. MeJA mediates both intra- and inter-plant communication to activate defense signaling cascades^9–11^. Its perception by cellular receptors leads to derepression of transcription factors and activation of genes involved in producing antioxidants, phytoalexins, and other secondary metabolites that enhance resistance^10,11^. As generally understood, MeJA signaling represents a finely tuned program that strengthens defense through extensive transcriptional reprogramming and cross-talk with other hormonal pathways^12–17^. However, despite substantial progress in elucidating transcriptional regulation by MeJA, its potential role in post-transcriptional regulation of gene expression, specifically regulating protein synthesis remains poorly understood^18,19^.

Regulation of protein synthesis (translational control) is fundamental to plant growth, development, and management of defense responses^20–22^. Translational control occurs majorly, but not exclusively, during the phase of initiation and involves the action of major signaling protein kinases, such as the general control non-derepressible 2 (GCN2), SNF-related kinase (SnRK), and the target of rapamycin (TOR)^20^. Among these, GCN2 has received significant attention in regulating translation under different types of abiotic, biotic, and xenobiotic stresses^23–31^. The diverse stress sensing/signaling by the GCN2 protein aptly attributes its role as one of the most important “stress sensor” kinase in plants^32–34^. In mammals and yeast, GCN2 protein is activated in response to amino acid deprivation and phosphorylates the α-subunit of the heterotrimeric eukaryotic translation initiation factor 2 (eIF2) on a conserved N-terminal serine residue. Phosphorylation of eIF2α (henceforth P-eIF2α) results in suppression of global translation while allowing the selective translation of stress-responsive mRNAs (e.g., *GCN4* in yeast). In plants, the GCN2-eIF2α module is activated in response to a wide variety of abiotic (e.g., cold, excess light, salt), biotic (e.g., bacterial, fungal pathogens), and xenobiotic factors (e.g., herbicides, cadmium, hydrogen peroxide)^26–28^. Specifically, Lokdarshi et al., 2020ab identified reactive oxygen species (ROS) as a key activator of the Arabidopsis GCN2-eIF2α module. Their work showed that certain stresses, such as excess light, cold, salt, and herbicides, all induce the accumulation of ROS and trigger rapid (within 10–30 min) activation of GCN2^26–28^. Given the rapid response nature of the GCN2-eIFα module and the activation signal as ROS, their findings highlight the potential of translational control via the GCN2-eIFα module to explain how plant cells can nimbly adjust to dynamic environments.

Here, we report that Arabidopsis eIF2α is phosphorylated by the GCN2 protein within 30 min of MeJA treatment. We show that light is essential for the activation of GCN2 in response to ectopic MeJA treatment, and the increase in P-eIF2α is attenuated by the application of a photosynthetic inhibitor and ROS quencher. As observed previously under numerous stress conditions, *loss-of*-*GCN2* knock-out mutants show severe growth defects under prolonged MeJA stress. However, both wild-type and *gcn2-1* mutant show a similar rate of protein synthesis under MeJA stress as evidenced by polysome profiling and puromycin incorporation assay. We also show that P-eIF2α is majorly dependent on the presence of the pan-eukaryotic GCN2 activator protein, general control non-derepressible 1 (GCN1), and *loss-of-GCN1* function is deleterious for plant health under MeJA stress. In summary, the current study supports the critical role of photosynthetic ROS and introduces GCN1 as an essential component of the Arabidopsis GCN2-eIF2α module activation and signaling in response to MeJA stress.

## Results

### Methyl jasmonate induces GCN2-dependent phosphorylation of eIF2α

**Fig. 1:**
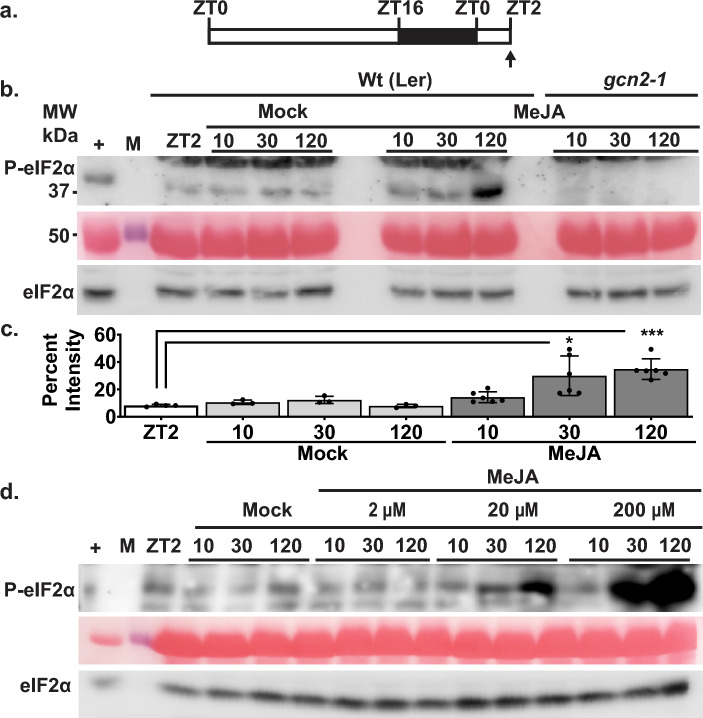
Methyl Jasmonate (MeJA) triggers GCN2-dependent P-eIF2α in a dose dependent manner. (**a**) Schematic of 16 h light (Zeitgeber time ZT0—ZT16) and 8 h dark (ZT16—ZT0) cycle. The black arrow indicates the time of sampling at ZT2 (before treatment) and the start of treatments. (**b**) Immunoblot showing the time course of P-eIF2α (top panel) and total eIF2α (bottom panel) in 12-days-old wild-type Landsberg (Wt (Ler)) and *gcn2-1* mutant (*gcn2-1*) seedlings treated with either Mock or 20 μM MeJA. (**c**) Quantification of P-eIF2α signal intensity from Fig. 1b. Error bars represent standard deviation of the mean from at least four independent experiments (Welch’s *t*-test *p-value = 0.0142, ***p-value = *0.003*). (**d**) Dosage treatment of Wt seedlings with 2, 20, and 200 μM MeJA as described in panel (a, b). For the panels b and d: Full length gels were cropped to the size represented in the figure. Panel b immunoblot used P-eIF2α antibody from Abcam; panel d from Cell Signaling. Original blots are presented in the Supplementary Fig. 6. The middle panel is a loading control showing the Rubisco large subunit (~ 55 kDa) in the respective P-eIF2α blots after Ponceau S staining; ( +) arbitrary amount of total protein extract from hydrogen peroxide treated Wt seedlings indicating unphosphorylated (eIF2α) or phosphorylated (P-eIF2α) protein (~ 38 kDa); (10, 30, 120) sampling time in minutes; (M) Molecular weight marker.

A previous report by Lageix et al., 2008 showed P-eIF2α as a read out of GCN2 activity after 4 h of MeJA treatment. Given that P-eIF2α levels increase significantly within 30–120 min under a wide variety of stresses^26–28^, we tested whether the activation of GCN2 under MeJA stress also followed a rapid activation model. Upon MeJA treatment, P-eIF2α levels increased significantly within 30 min and remained elevated up to 120 min, compared to ZT2, in the wild-type Arabidopsis seedlings (Fig. 1 a-c). The homozygous *gcn2* mutant (*gcn2-1*) failed to show P-eIF2α signal, suggesting that GCN2 protein is essential for rapid P-eIF2α under MeJA. In addition to the reliance on time, the GCN2 activity was responsive to increasing MeJA concentrations (Fig. 1 d). Treatment with 200 μM MeJA showed higher P-eIF2α versus 2 μM and 20 μM MeJA at both the 30- and 120-min time intervals. In all the above treatments, the total eIF2α levels remain unchanged, indicating that GCN2-dependent P-eIF2α serves as a potential signaling mechanism for MeJA in Arabidopsis.

### Dark and ROS quenchers mitigate GCN2 activity under MeJA stress

**Fig. 2:**
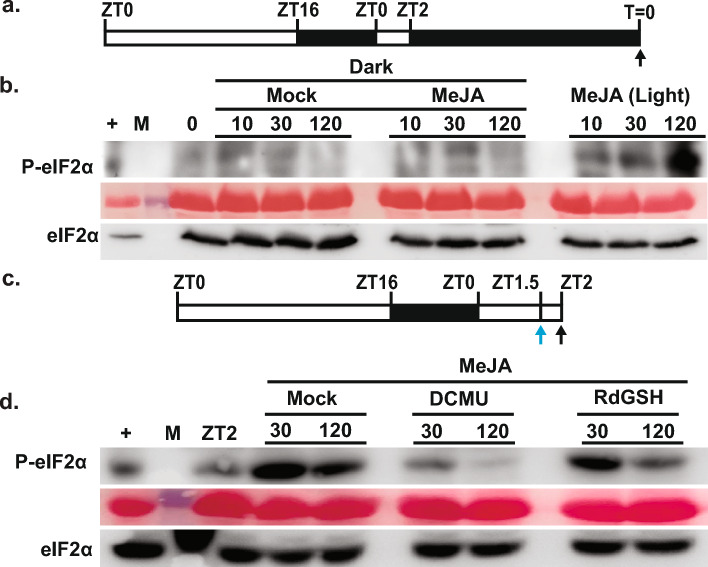
Dark and ROS quenchers attenuate GCN2 activation by MeJA. (**a**) Schematic of 24 h dark acclimation to 12-days-old Wt (Ler) seedlings. Black arrow indicates the time of sampling at T = 0 (at the end of 24 h dark period) and represents the time of reagent application. (**b**) P-eIF2α in Wt seedlings treated with either Mock or 20 μM MeJA under dark as described in panel A. MeJA treatment under light performed as described in Fig. 1. (**c**) Growth regime showing the time of treatment with either mock or ROS quenchers, thirty minutes prior to MeJA treatment (ZT1.5/blue arrow). Black arrow indicates the time of sampling at ZT2 (end of ROS quencher treatment) and represents the time of MeJA application. (**d**) P-eIF2α in Wt seedlings treated with either Mock, or 1 mM reduced glutathione (RdGSH), or 30 μM (3-(3,4-dichlorophenyl)-1,1-dimethylurea) (DCMU) thirty minutes prior to 20 μM MeJA treatment under light as described in panel c. For the panels b and d: Full length gels were cropped to the size represented in the figure. Original blots are presented in the Supplementary Fig. 6. For detailed legend to panel b and d, please see Fig. 1.

Under numerous stresses including excess light, cold, salt, and herbicide, the phosphorylation of eIF2α has been shown to be dependent on light^26,27^. Moreover, the treatment with antioxidants or photosynthetic inhibitors alleviates Arabidopsis GCN2 activation across these different stress conditions^26,27^. To investigate whether MeJA-triggered GCN2 activation is also under the control of light, we acclimated the wild-type Arabidopsis seedlings for 24 h in dark (Fig. 2 a). Exposure of the dark-adapted seedlings to MeJA failed to illicit significant P-eIF2α signal compared to treatment under light (Fig. 2 b). Moreover, pre-treatment of wild-type seedlings with an antioxidant, reduced glutathione (RdGSH), or a photosynthetic inhibitor, 3-(3,4-dichlorophenyl)-1,1-dimethylurea (DCMU), suppressed the GCN2 activation significantly in response to MeJA compared to the mock (DMSO) pre-treatment (Fig. 2 c-d). Building on Zhang and Xing’s (2008) findings that MeJA induces accumulation of ROS in chloroplasts within 3 h, we also detected an increase in the H_2_O_2_ levels in response to MeJA treatments as early as 30 min and 2 h compared to ZT2 (Supplementary Fig. 1). Combined, these results underscore the importance of chloroplast function and ROS in activating the GCN2 protein under MeJA-induced stress.

### Loss-of-GCN2 mutant seedlings are sensitive to MeJA stress

**Fig. 3:**
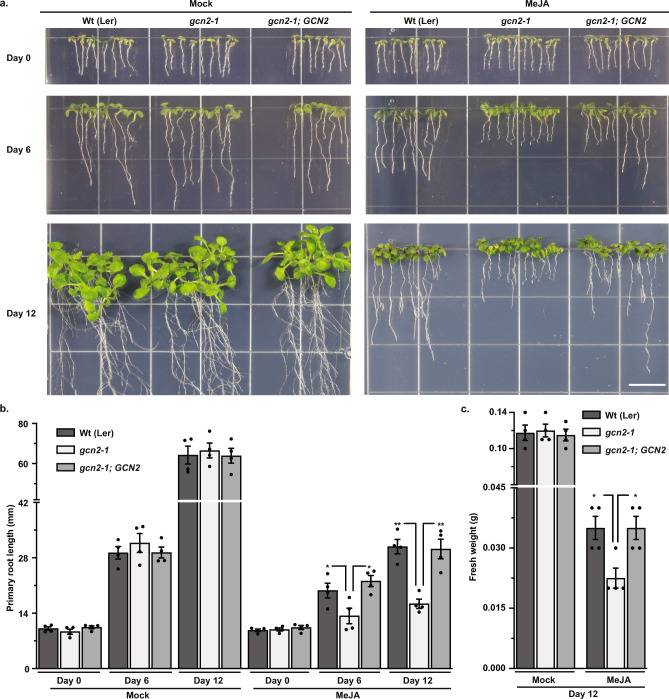
Loss of *gcn2-1* renders increased sensitivity towards MeJA stress. **(a)** Top panel: Representative images of 3-day-old Wt (Ler), *gcn2-1* mutant and *GCN2* complementation (*gcn2-1; GCN2*) seedlings grown under a 16 h light / 8 h dark long day cycle. Seedlings were transferred on plant media with Mock or 20 μM MeJA on Day 0. Middle and Bottom panel: Same seedlings after six (Day 6) and twelve days (Day 12) of growth. Scale bar (white) is 2.54 cm. **(b)** Primary root length in millimeters (mm) of all seedlings on Day 0, 6 and 12 from panel a. Error bars represent standard error mean of four biological replicates (Welch’s *t*-test Day 6 Wt vs gcn2-1 *p-value = 0.0267, gcn2-1; GCN2 vs gcn2-1 *p-value = *0.0127*; Day 12 Wt vs gcn2-1 **p-value = 0.0010, gcn2-1; GCN2 vs gcn2-1 **p-value = *0.0060*). (c) Fresh weight in grams (g) of all seedlings on Day 12 from panel a. Error bars represent standard error mean of four biological replicates (Welch’s *t*-test *p-value = *0.0175*).

*GCN2* is essential to the plant survival/adaption responses, and the loss of *GCN2* function renders the plant sensitive toward different types of stresses, such as continuous light, excess light, salt, cold, and herbicides^26,27,29,35^. To determine the role of *GCN2* under MeJA stress, we tested the phenotypic response of two Arabidopsis *GCN2* homozygous mutants: *gcn2-1*^29,30^ in the Landsberg ecotype and *gcn2-2* in the Columbia ecotype^36^. On the mock (DMSO) growth medium, *gcn2-1* seedlings exhibit similar growth as the wild-type and the *GCN2* complementation line, *gcn2-1; GCN2*^30^ (Fig. 3 a). However, when grown on MeJA-containing media, *gcn2-1* seedlings show significant reduction in primary root growth and overall fresh weight compared to wild-type and the *GCN2* complementation line at day 6 and day 12 of the stress treatment (Fig. 3 a-c). Additionally, we assessed the levels of anthocyanin, a flavonoid pigment that is known to mitigate ROS accumulation as part of the plant’s stress resilience program^37^. Moreover, at day 12, wild-type seedlings exhibited significantly higher anthocyanin levels versus *gcn2-1* grown on media with 20 μM MeJA, and these levels further increased with 100 μM MeJA treatment (Supplementary Fig. 2). These findings align with the observation in the Columbia ecotype, where *gcn2-2* seedlings also exhibit significant primary root growth retardation and lower fresh weight versus wild-type on MeJA-containing media (Supplementary Fig. 3). Combined, our results highlight the essential role of *GCN2* in managing survival responses towards MeJA stress in Arabidopsis.

### Rate of global protein synthesis appears similar in wild-type and gcn2 mutant in response to MeJA stress

**Fig. 4:**
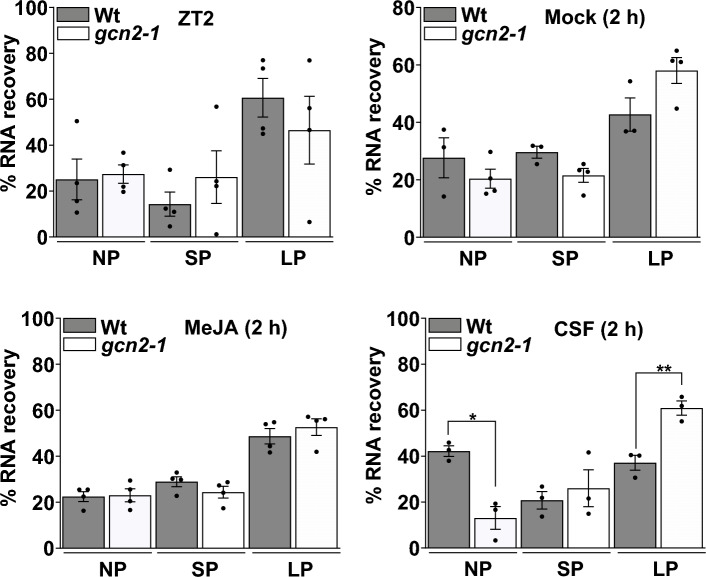
Wild-type and *gcn2-1* mutant show similar translation state under MeJA stress. Histograms showing percent RNA recovery from sucrose gradient fractions of 12-day-old wild-type Landsberg (Wt (Ler)) and *gcn2-1* mutant seedlings at ZT2 and after 2 h of treatment with Mock, or 20 μM MeJA, or 0.6 μM chlorosulfuron (CSF). NP = Non-Polysomal; SP = Small Polysomal; LP = Large Polysomal. Error bars represent standard error mean of four biological replicates for ZT2, Mock, MeJA and three biological replicates for CSF treatment (Welch’s *t*-test *gcn2-1* vs Wt(Ler) NP *p-value = *0.0150*; LP **p-value = *0.0061*).

In yeast and mammals, amino acid starvation triggers the activation of the GCN2 protein, which results in down-regulation of cytosolic protein synthesis to conserve energy and adapt to nutrient depletion^38,39^. This phenomenon appears to be partially conserved in plants, as demonstrated by Lageix et al. (2008) and Lokdarshi et al. (2020a), who observed elevated large polysome content in *gcn2-1* mutants compared to wild-type seedlings under chlorsulfuron (CSF) stress, suggesting active translation in the mutant despite being under stress. Based on this observation, we hypothesized that similar changes in the polysome profiles of the wild-type and *gcn2-1* mutant seedlings would be evident under MeJA stress. To investigate the role of GCN2 in regulating global translation under MeJA stress, we assessed the rRNA (18S and 28S) recovery from the sucrose gradient fractions using agarose gel electrophoresis (Supplementary Fig. 4). Both wild-type and *gcn2-1* seedlings showed similar rRNA recovery of the non-polysomal (NP), small polysomal (SP), and large polysomal (LP) fractions under mock (DMSO) and MeJA treatment (Fig. 4). Additionally, a puromycin assay revealed no statistically significant differences in the anti-puromycin hybridization signal between wild-type and *gcn2-1* under both mock (DMSO) and MeJA treatment, indicating similar profiles of translating ribosomes (Supplementary Fig. 5). These results identify MeJA stress as an activator of the Arabidopsis GCN2-eIF2α module without inducing a detectable global translational repression.

### GCN1 is essential for the activation of GCN2 and maintenance of adequate growth under MeJA stress

**Fig. 5:**
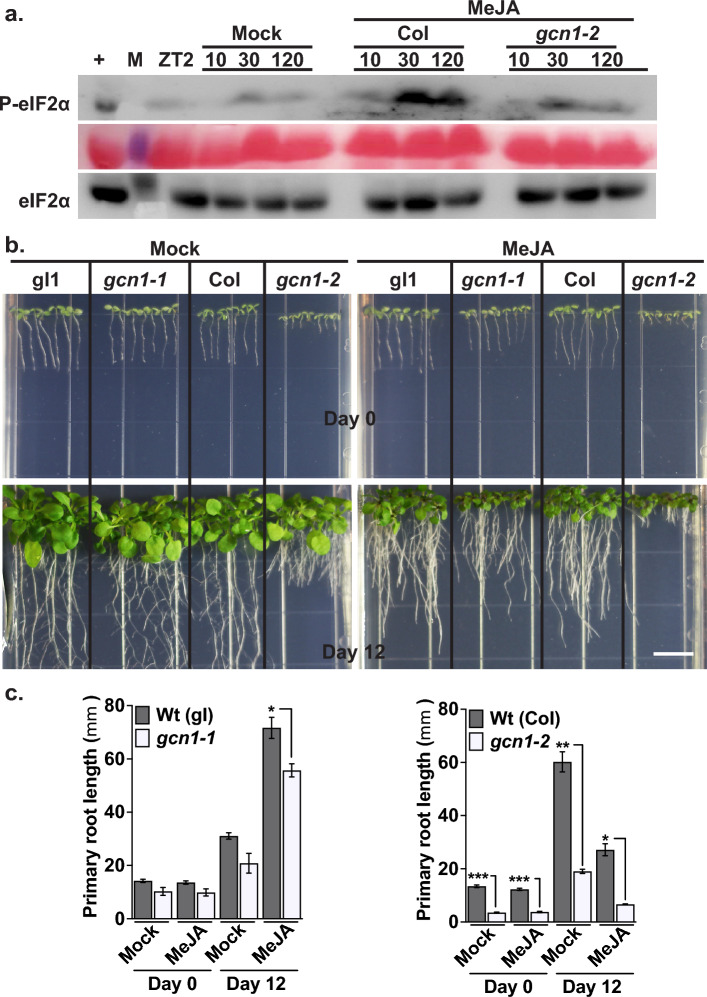
*GCN1* is essential for P-eIF2α and maintenance of growth under MeJA stress. (**a**) Top panel: Immunoblot showing the time course of P-eIF2α in 12-days-old wild-type Columbia (Col) and *gcn1-2* mutant (*gcn1-2*) seedlings treated with either Mock or 20 μM MeJA. For detailed legend to panel a, please see Fig. 1. Original blots are presented in the Supplementary Fig. 7. (**b**) Top panel: Representative images of 3-day-old wild-type Columbia glabrous (gl1), *gcn1-1* mutant (*gcn1-1*), wild-type Columbia (Col) and *gcn1-2* mutant (*gcn1-2*) seedlings grown under long day cycle. Seedlings were transferred on plant media with Mock or 20 μM MeJA on Day 0. Bottom panel: Same seedlings after twelve days (Day 12) of growth. Scale bar (white) is 2.54 cm. (**c**) Primary root length in millimeters (mm) of all seedlings on Day 0 and Day 12 from panel b. Error bars represent standard error mean of three biological replicates (Welch’s *t*-test Day 12 Wt (gl) vs gcn1-1 *p-value = *0.0186*; Day 0 Mock Wt (Col) vs gcn1-2***p-value = *0.003*, MeJA ***p-value = *0.009*; Day 12 Mock **p-value = *0.0064*, MeJA *p-value = *0.0112*).

In yeast, the interaction between GCN1 and GCN2 is essential for the ability of GCN2 to phosphorylate eIF2α under various stresses^40^. Specifically, GCN1 is required for the activation of GCN2 during amino acid starvation, exposure to Ultraviolet (UV) light, and H_2_O_2_-induced oxidative stress^41–43^. Consistent with its role in yeast, Arabidopsis GCN1 has been shown to be essential for P-eIF2α in response to numerous stresses including chlorosulfuron, exposure to UV, endoplasmic reticulum (ER) stress, cold, and macronutrient (Nitrogen or Phosphorus) deficiency^44–46^, supporting the role of GCN1 as key regulator of physiological responses to various types of stresses in Arabidopsis. To investigate the role of GCN1 in MeJA-induced P-eIF2α, we hypothesized that the Arabidopsis *gcn1* mutant would exhibit low P-eIF2α signal under MeJA stress. Furthermore, we hypothesized that the *gcn1* mutants would exhibit phenotypic abnormalities, similar to the *gcn2* mutant seedlings under prolonged MeJA stress. As expected, the *gcn1* mutant (*gcn1-1*) show reduced P-eIF2α levels compared to wild-type at both 30- and 120-min time intervals (Fig. 5 a), confirming that GCN1 is important for GCN2-dependent P-eIF2α under MeJA stress. Additionally, *gcn1-1* mutants exhibited significant growth retardation and reduced primary root length on MeJA-containing medium. This phenotype was also observed in a second loss-of-function allele, *gcn1-2*, which displayed severe growth defects and similar abnormalities under MeJA stress (Fig. 5 b-c). These results suggest that GCN1 is crucial for both P-eIF2α, and also for the maintenance of normal growth and development, especially under MeJA stress.

## Material and methods

### Plant materials and growth conditions

Seeds of the following *Arabidopsis thaliana* ecotypes and mutants were sterilized and stratified at 4 °C for 2 days on ½-strength Murashige-Skoog (MS) salt plant medium (MP Biomedicals, Cat# 2,633,024) containing 0.65% Phytoagar (Bioworld, Cat # 40,100,072–2).

- Wild-type Landsberg *erecta* (Ler-0), Columbia-0 (Col_0) and Columbia glabrous (Col (gl1));
- Homozygous *gcn2-1* mutant in Ler-0 (Genetrap line GT8359)^29,30^ and *gcn2-1;GCN2* complementation with *GCN2* under native promoter^30^;
- Homozygous *gcn2-2* mutant in Col_0 (SALKseq_032196)^36^;
- Homozygous *gcn1-1* mutant in Col (gl1)^44^;
- Homozygous *gcn1-2* mutant in Col_0^44,47^.

Seeds were germinated and grown under standard long-day cycle of 16 h light (white light, 80 ± 10 µE m^-2^ s^-^) / 8 h dark at 22 °C and 50% humidity.

### Stress treatments

For stress treatments under light, 12-days-old horizontally (roots inside the media) grown seedlings were sprayed 10–12 times from a distance of 2 inches with the following reagents. After 20 s, excessive reagent was drained off and plates were wrapped with 3 M pore surgical tape (Fisher Scientific, Cat# 22–355-000). Mock – 0.01% (v/v) Dimethyl Sulfoxide (DMSO) (Fisher Scientific, Cat# D128-500) or double deionized sterile water; MeJA – Methyl Jasmonate (Millipore Sigma, Cat# 392,707) in 0.01% (v/v) DMSO; CSF – Chlorosulfuron (Millipore Sigma, Cat# 34,322) in 0.01% (v/v) DMSO; DCMU – (3-(3,4-dichlorophenyl)-1,1-dimethylurea) (Fisher Scientific, Cat# AAL0298618) in 0.01% (v/v) DMSO; RdGSH – Reduced Glutathione (Millipore Sigma, Cat# 70–18-8) in deionized water.

For pre-treatment with DCMU or RdGSH, seedlings were sprayed at ZT1.5 (ZT = Zeitgeber time; 1.5 h after lights-on) and incubated for 30 min before stress treatments at ZT2 as described above. Stress treatments under dark were performed with seedlings after dark-acclimatation for 24 h, i.e., starting two hours after lights-on (ZT2). Treatment with different reagents was performed as described for stress under light. Dark sampling was performed under safe green light.

### Phenotype characterization (Primary root length and fresh weight measurement)

Three-days-old vertically (roots on top of the media) grown seedlings were transferred onto either mock or MeJA containing media (Day of the transfer = Day 0) and grown under long-day cycle for the period indicated in the respective figures. Photographs of the seedings were taken with a digital camera (Canon 60D). The primary root length was measured using ImageJ (ver.1.54 g; http://rsb.info.nih.gov/ij/index.html) and fresh weight was analyzed by weighing the seedlings at the end of the stress treatments. All statistical analysis was performed using GraphPad Prism (ver. 5 or 10; GraphPad Software, Inc).

### Total protein extraction, P-eIF2α /GCN2 activation assay and Quantification of P-eIF2α signal intensity

Total protein was extracted as described previously (Zhang et al., 2008) with slight modification. Briefly, 50–150 mg of 12-days-old horizontally grown seedlings were stored in a pre-filled bead mill tube (Fisher Scientific, Cat# 15–340-151) and flash frozen in liquid N_2_. Tubes were stored at -80^0^C prior to immunoblot analysis. For total protein extraction, 80–120 μl of freshly prepared extraction buffer containing 25 mM Tris–HCl (pH 7.5), 75 mM NaCl, 1 M Urea, 5% (v/v) glycerol, 0.5 mM EDTA, 0.5 mM EGTA, 2 mM DTT, 2% (w/v) insoluble Polyvinylpyrrolidone (Sigma-Aldrich, Cat# P-6755), supplemented with 1X Protease and Phosphatase inhibitor cocktail (Thermo-Fisher; Cat# PIA32959) was added into the tubes. Seedlings were ground using a bead mill (Fisher Scientific, Cat# 15–340-163) as per manufacturer’s instructions and total protein content was quantified by Bradford assay (Thermo-Fisher, Cat# 23,236).

The P-eIF2α/GCN2 activation assay was performed by immunoblot analysis as described in Lokdarshi et al., 2020ab. P-eIF2α was detected with the Cell Signaling Technology Phospho-eIF2α (Ser51) rabbit polyclonal antibody (Cat# 9721S) or anti-eIF2S1 (phospho-S51) antibody (Abcam, Cat# 32,157) and total eIF2α was detected with a rabbit polyclonal eIF2α antibody (a gift from Dr. Karen Browning, University of Texas, Austin) as described in Lokdarshi et al., 2020ab. Chemiluminescent images were capture with GE ImageQuant LAS-4000 Multi-Mode Imager using ImageQuant LAS-4000 software.

For quantification of P-eIF2α signal intensity, raw images of the immunoblots were analyzed using the default gel analysis tool in the ImageJ program version 1.54 g. Peak areas from the intensity profile plots were copied into Microsoft Excel. The total peak area for each blot was set as 100% signal intensity. Then, each time point’s peak area was divided by this total to calculate its percent signal intensity. All statistical tests for significance were performed using GraphPad Prism version 5 or 10.

### Polysome Profiling and ribosomal RNA analysis

For the polysome profiling, 12-days-old horizontally grown seedlings were flash frozen in liquid N_2_ and stored at -80℃. Seedlings were ground in liquid N_2_ using pre-chilled pestle and mortar and polysome profiling was performed as described previously in Missra et al., 2015^48^ and Lokdarshi et al., 2020ab. For fractionation, 900 μl of the gradient was manually aspirated and transferred into a fresh 2 ml tube for RNA extraction as described in Missra & von Arnim 2014^49^. RNA quality/quantity was assessed by a Nanodrop Spectrophotometer (Thermo Scientific, Cat# 13–400-519) and statistical analysis of percent RNA recovery was performed with GraphPad Prism Version 10.

Based on the agarose gel analysis of rRNA (18S and 25S) (see Supplementary Fig. 4), three fractions of RNAs were generated: NP (non-polysomal) with either none, SP (small polysomal) with individual ribosomal subunits (40S, 60S) or considering one-two ribosomes (80S) per mRNA, and the LP (large polysomal) fractions representing multiple ribosomes per mRNA.

### Puromycin assay

The rate of global protein synthesis with puromycin (PU) was performed as described previously in Llabata 2019 with slight modifications. Briefly, 10-days-old horizontally grown seedlings were treated with either mock or MeJA for 2 h. At the end of the stress treatments, 15 ml of 65 µg ml^-1^ Puromycin Dihydrochloride (Fisher Scientific, Cat# AC227422500) was added to the plates and the seedlings were further incubated for 2 h under the same growth conditions. Seedlings were transferred on a dry paper towel to remove excessive liquid before weighing and flash frozen in liquid N_2_ and stored at -80℃. Sample processing and immunoblot experiment was performed as described in Llabata 2019. The rate of translation was determined by measuring the signal intensities of all the dots from the dot blot assay using ImageJ (ver.1.54 g; http://rsb.info.nih.gov/ij/index.html) and statistical analysis was performed using GraphPad Prism Version 10.

### Hydrogen peroxide quantification

Quantification of in vivo H_2_O_2_ levels was performed as described in Lokdarshi et al., 2020ab using the Amplex Red kit (Thermo-Fisher, Cat# A22188). Briefly, 30 mg of 12-days-old seedlings were flash frozen in liquid N_2_ and ground with a plastic pestle for 2 min until a homogenous powder was visible. Tissue powder was resuspended in 100 μl of 1X reaction buffer (Amplex Red kit) and centrifuged at 17,000 × g 4 ºC for 2 min. The supernatant was used for H_2_O_2_ measurements as per manufacturer’s protocol. Absorbance at 590 nm was measured on the SpectrMax M5e (Molecular Devices, VWR Cat# 89,212–400) plate reader.

### Anthocyanin quantification

Quantification of anthocyanin was performed as described previously in Nakata & Ohme-Takag 2014^50^ with seedlings at Day 12 of the phenotype characterization. Briefly, 30–50 mg of seedlings were flash frozen in liquid N_2_ and grinded in a 1.5 ml tube using plastic pestle. Pulverized tissue powder was resuspended in 5 volumes (based on fresh weight) of freshly prepared extraction buffer (45% (v/v) Methanol, 5%(v/v) Acetic acid). After centrifuging the tube at 12,000 × g for 5 min at room temperature, supernatant was transferred to a fresh 1.5 ml tube and recentrifuged to clear remaining cell debris. Anthocyanin content was measured by transferring 50 μl of the supernatant into a 96 well clear plate and recording absorbance at 530 nm and 657 nm on the SpectraMax M5e plate reader. Statistical analysis was performed using GraphPad Prism Version 10.

## Discussion

Lageix et al. (2008) demonstrated P-eIF2α at 4 h and 12 h post-MeJA treatment, while our current study further shows that MeJA-induced stress not only rapidly triggers P-eIF2α (within 30 min), but also requires the presence of light for this activation. Moreover, the reduction of P-eIF2α signal in seedlings pre-treated with photosynthetic inhibitor DCMU, or the ROS quencher RdGSH, are consistent with our earlier findings regarding rapid Arabidopsis GCN2 activation in response to various stressors and the dependence on chloroplast function^26,27^. Considering the chloroplast’s role as the primary producer of ROS during photosynthesis and its transmission of redox and ROS signals to the cytosol for rapid adjustments in transcription and translation^51,52^, our findings support the involvement of chloroplastic ROS in GCN2 activation. Although the exact biochemical mechanism(s) underlying this common activation pathway remains to be fully elucidated, starvation of amino acids upon exposure to MeJA, resulting in the accumulation of uncharged tRNA within 10–30 min, are unlikely to be the primary causes of rapid GCN2 activation. The early activation of the kinase following MeJA application likely stems from either direct activation by ROS or other GCN2 activators that are responsive to rapid ROS signaling. While we do not discount the conventional model of GCN2 activation by uncharged tRNA, we propose an alternative scenario where ROS serves as a quick activation ligand followed by the involvement of both ROS and uncharged tRNA. Dissecting these hypotheses will offer deeper insights into the activation mechanisms of GCN2 under MeJA stress and other ROS-related stresses.

The GCN1 protein is a crucial activator of GCN2, and this function of GCN1 seems to be evolutionarily conserved across species^53^. In Arabidopsis, GCN1 is necessary for translational arrest under cold stress by interacting with GCN2, which leads to the phosphorylation of eIF2α^44^. Our observation of a strong reduction in P-eIF2α levels in the *gcn1-2* mutant versus wild-type in response to MeJA treatment reinforces the essential role of GCN1 in GCN2 activation, as shown recently by Cui et al. under macronutrient (N or P) starvation^46^. Their study showed that nutrient deprivation induces ROS accumulation, which in turn activates the GCN1-GCN2 pathway to promote P-eIF2α. In our study, MeJA induces oxidative stress primarily through the chloroplast-derived ROS, which likely activates the GCN1-GCN2 signaling cascade.

The difference in the extent of P-eIF2α signal loss between the two studies, specifically our detection of residual phosphorylation in the *gcn1-2* under MeJA stress versus the complete absence in *gcn1-1* under N starvation shown by Cui et al., could reflect the involvement of additional signaling components or compensatory mechanisms during rapid MeJA-induced ROS stress that under nutrient deprivation are absent and/or less active. Nonetheless, both the studies converge on our previously proposed model in which chloroplastic-ROS serves as a key upstream signal linking environmental stress to translational control via the GCN2-eIF2α signaling module. Finally, the phenotypic abnormalities observed in the *gcn1* mutant reinforce the critical role of GCN1 in activating the GCN2-eIF2α signaling to counteract MeJA-induced stress. While the mechanistic link between MeJA exposure, chloroplast-derived ROS, and GCN1-mediated activation of the GCN2 remain to be fully elucidated, our results highlight the conserved role of GCN1 as a GCN2 activator protein in plant stress.

Previous studies have demonstrated a clear sequence of events with herbicides inhibiting amino acid synthesis, where GCN2 activation by the synthetic non-natural agents such as chlorosulfuron, in the presence of light, leads to P-eIF2α, subsequent global translational repression, and hypersensitivity in *gcn2* mutants^26,27^. However, the regulation of translation in response to more physiologically relevant, natural abiotic stimuli such as cold, salt, and excess/high light appears less straightforward. Our findings indicate that while GCN2 protein rapidly phosphorylates eIF2α following MeJA treatment, there’s no detectable global translational repression within 2 h. Similar patterns have been reported under other ROS-related stresses, where P-eIF2α occurs without an immediate or corresponding suppression of global translation^26,27^. Furthermore, high light stress, despite eliciting sensitivity in the *gcn2* mutants does not induce GCN2-dependent translational repression^26^. These observations suggest that P-eIF2α is not universally coupled to translational repression, and conversely, not all instances of translational repression depend on P-eIF2α. Given that ROS accumulation is localized in Arabidopsis leaves exposed to high light or herbicide stress^26,27^, it is plausible that retrograde control of translation may be a potent mechanism for immediate spatial and temporal adjustment of the cytosolic translation and chloroplast proteome. Furthermore, it is hypothesized that retrograde control of translation under high light could allow for spatially heterogeneous protein synthesis^52^. This molecular arrangement would mean preferential loading of transcripts that directly code for repair and photosynthesis related proteins thereby supporting a faster translational retrograde circuit. How the GCN2-eIF2α module functions within the proposed spatiotemporal translation control under MeJA induced ROS stress remains to be tested.

Taken together, our study reveals new information that Arabidopsis MeJA signaling involves the highly conserved GCN2-eIF2α module working under the command of chloroplastic ROS and GCN1. Despite *gcn2* mutant displaying similar in vivo translation status as the wild-type under short MeJA challenge, it exhibits heightened sensitivity to prolonged MeJA stress in the phenotypic assay. These findings bolster the proposed model of chloroplastic ROS triggering rapid activation of cytosolic GCN2, regulation of translation under diverse abiotic stresses and long-term adjustments to energy management. Future research should elucidate the biochemical and molecular events leading to GCN2 activation under MeJA and ROS related stresses, shedding light on the elusive integrated stress response pathway in plants. Furthermore, investigating the regulation of global translation versus stress-specific mRNA targets warrants further exploration for deeper understanding of the role of GCN2 as a stress sentinel kinase in plants.

## Supplementary Information

Supplementary Information.

## Data Availability

All data generated or analyzed during this study are included in this published article (and its Supplementary Information files).
