# Supplementary material for: GCN2 is activated by methyl jasmonate through GCN1 and reactive oxygen species in Arabidopsis thaliana
Source: Sci Rep. 2026 Jan 13;16:2918. doi: 10.1038/s41598-025-32799-w (PMC12827998; doi:10.1038/s41598-025-32799-w)
Supplement: Supplementary file 1 — Supplementary Information. [file 41598_2025_32799_MOESM1_ESM.pdf]

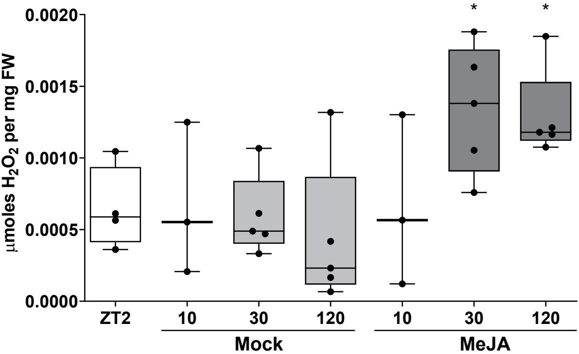

Supplementary Figure 1. Methyl Jasmonate stress triggers accumulation of hydrogen peroxide (H_2O_2). Relative H_2O_2 levels in 12-days-old wild-type Landsberg seedlings treated with either Mock or 20 µM MeJA as indicated in Fig. 1. Error bars represent standard error mean of at least three biological replicates (Welch's *t*-test 30 min **p-value = 0.0265; 120 min *p-value = 0.0149*). ZT = Zeitgeber; 10, 30 and 120 = Time of sampling in minutes after treatment.

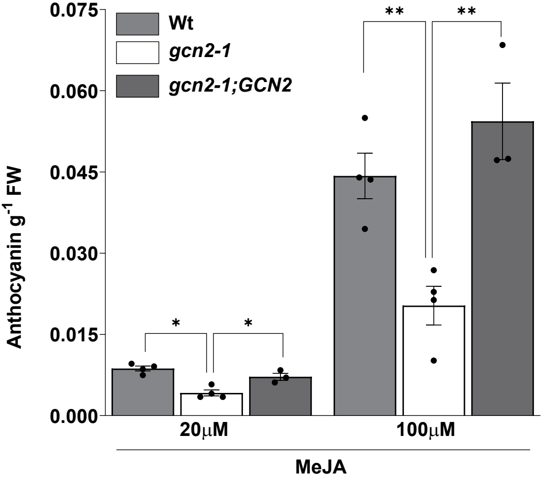

Supplementary Figure 2. *gcn2-1* mutant shows reduced accumulation of anthocyanin under MeJA stress. Anthocyanin content in wild-type Landsberg (Wt (Ler)), *gcn2-1* mutant (*gcn2-1*) and *GCN2* complementation (*gcn2-1; GCN2*) seedlings after twelve days of growth on either 20 μM or 100 μM MeJA containing plant media. Treatments were performed as described in Fig. 3. Error bars represent standard error mean of at least three biological replicates (Welch's *t*-test **p-value = 0.0285; **p-value = 0.0062*). FW = Fresh Weight.

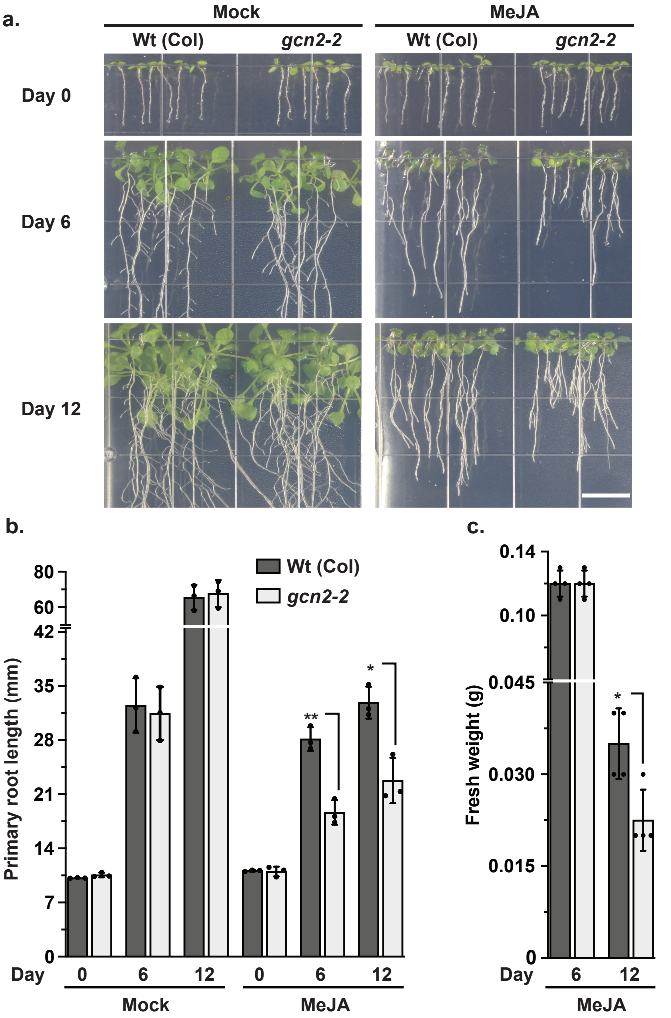

Supplementary Figure 3. *gcn2-2* mutant shows higher sensitivity towards MeJA stress.

(a) Top panel: Representative images of 3-day-old wild-type Columbia (Wt (Col)) and *gcn2-2* mutant seedlings grown under long day cycle. Seedlings were transferred on plant media with Mock or 20 μM MeJA on Day 0. Middle and Bottom panel: Same seedlings after six (Day 6) and twelve days (Day 12) of growth. Scale bar (white) is 2.54 cm.

(b) Primary root length in millimeters (mm) of all seedlings on Day 0, 6 and 12 from panel a. (Welch's *t*-test Day 6 ***p-value = 0038;* Day 12 **p-value = 0.0107*).

(c) Fresh weight in grams (g) of all seedlings on Day 12 from panel a. (Welch's *t*-test **p-value = 0.0175*).

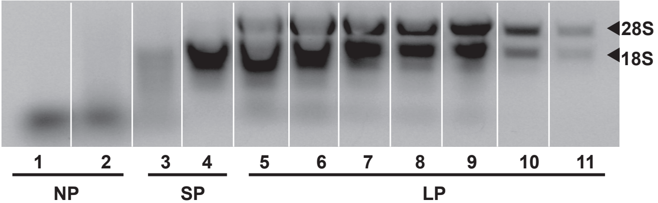

Supplementary Figure 4. Agarose gel showing ribosomal RNA profile. Representative image of ethidium bromide stained agarose gel showing the distribution of 18S (40S subunit) and 28S (60S subunit) ribosomal RNA in the 11 fractions from sucrose gradient (please refer to Fig. 4). Numbers 1-2 correspond to non-polysomal (NP), 3-4 to small polysomal (SP) and 5-11 to large polysomal (LP) fractions.

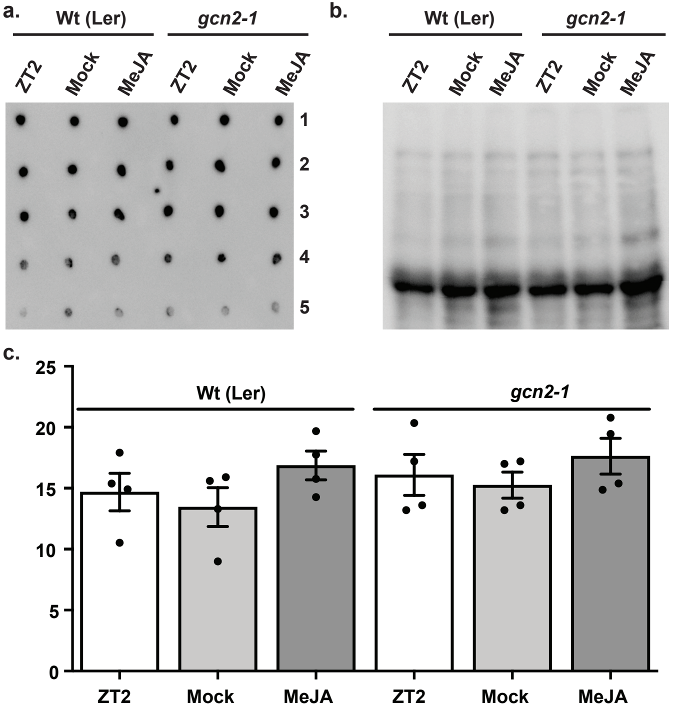

Supplementary Figure 5. Wt and *gcn2-1* mutant show similar rate of protein synthesis under MeJA stress.

(a) Dot blot analysis with total protein extracts of puromycin (PU) treated wild-type Landsberg (Wt (Ler)) and *gcn2-1* mutant (*gcn2-1*) seedlings at ZT2 and after 2 h of treatment with either Mock or 20 µM MeJA. The numbers on right (1-5) represents serial dilution of the total protein extract. PU incorporation was detected with anti-PU antibody.

(b) Anti-PU immunoblot showing PU incorporation in Wt and *gcn2-1* total protein extract after separation on 10% (w/v) sodium dodecyl sulfate polyacrylamide gel and transfer to Polyvinylidene difluoride membrane.

(c) Quantification of dot blot signal intensity shown in panel a. Error bars represent standard error of mean from four dilutions as shown in panel (a).

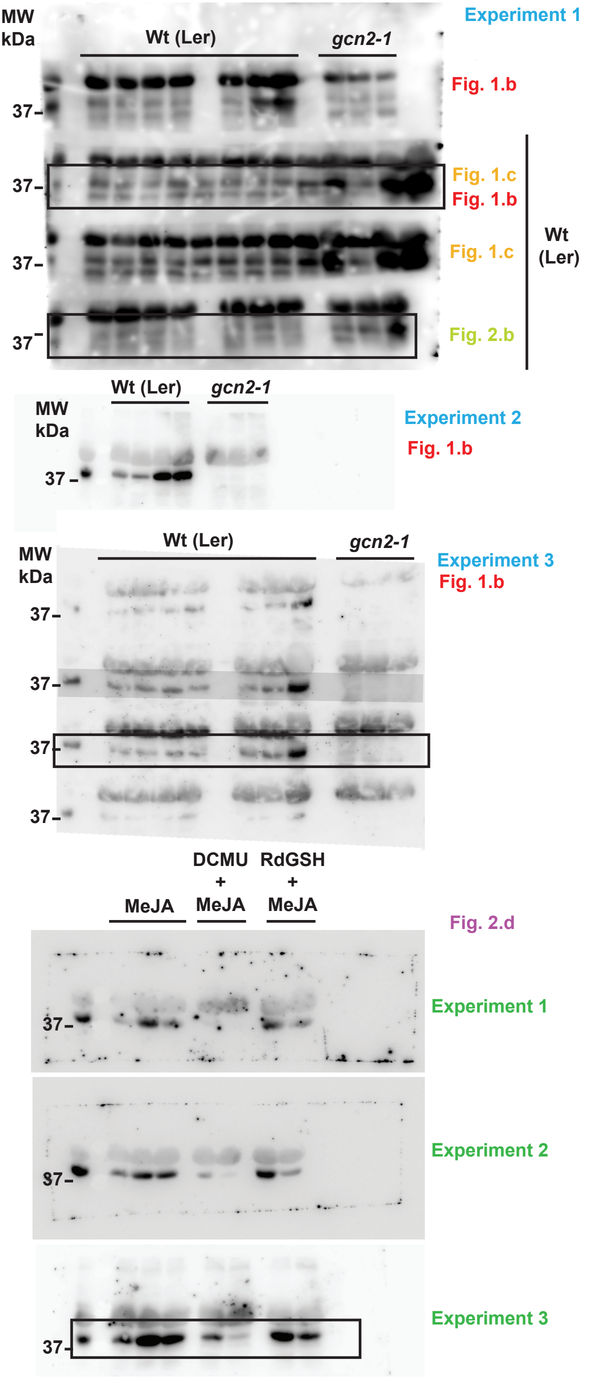

Supplementary Figure 6. Original immunoblot images showing the detection P-eIF2α. Each independent experiment (biological replicate) is labeled and color-coded to correspond with the respective figures in the main manuscript. Black boxes indicate the specific blot regions included in the respective final figure panels. The P-eIF2α signal in the top blot was detected using an antibody from Cell Signaling Technology (Cat# 9721S), while the remaining blots were detected using an antibody from Abcam (Cat# 32157). Quantificaiton of P-eIF2α signal shown in Fig.1 c is based on densitomteric analysis from the red colored labeled blots in Fig. 1. b.

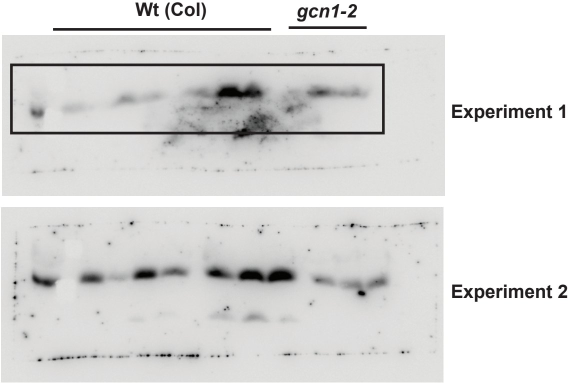

Supplementary Figure 7. Original immunoblot images showing the detection P-eIF2α from two independent experiments (Experiment 1 and Experiment 2), corresponding to Fig. 5a in the main manuscript. The black box outlines the specific region of the blot that was used in the final figure. The type of antibody used for detection is Abcam (Cat# 32157).

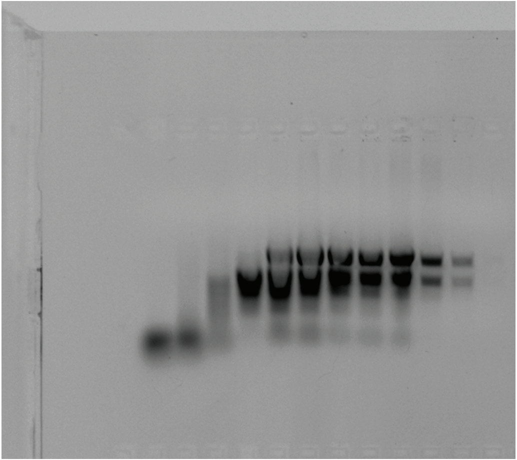

Supplementary Figure 8. Original agarose gel corresponding to Supplementary Figure 4.

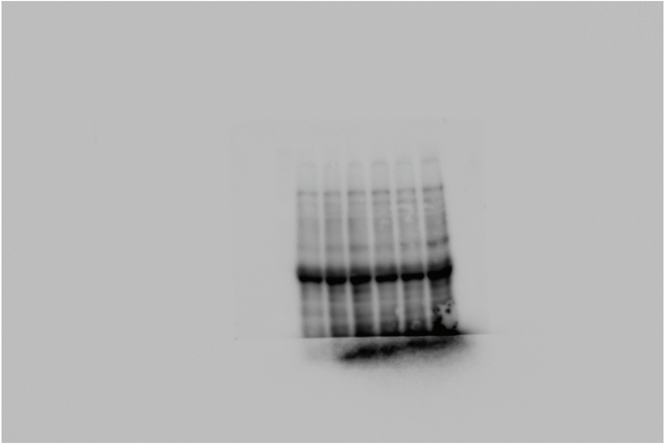

Supplementary Figure 9. Original blot corresponding to Supplementary Figure 5B.
